# Biochar of hazelnut shell mitigates cadmium toxicity in forage soybean

**DOI:** 10.3389/fpls.2025.1694806

**Published:** 2025-10-24

**Authors:** Sedat Severoğlu, Tuba Karabacak, Abdullah Yazıcı, Halit Aktaş, Nilay Çerit, Melek Ekinci, Mehmet Kerim Güllap, Metin Turan, Ertan Yildirim

**Affiliations:** ^1^ Department of Field Crops, Faculty of Agriculture, Atatürk University, Erzurum, Türkiye; ^2^ Department of Crop Production and Technologies, Muş Alparslan University, Muş, Türkiye; ^3^ Department of Horticulture, Faculty of Agriculture, Atatürk University, Erzurum, Türkiye; ^4^ Department of Agricultural Trade and Management, Faculty of Economy and Administrative Sciences, Yeditepe University, Istanbul, Türkiye

**Keywords:** biochar, biochemistry, cadmium stress, forage soybean, physiology

## Abstract

Cadmium (Cd) contamination threatens plant growth by disrupting physiological and biochemical processes, leading to reduced biomass and nutrient imbalances. Biochar, a carbon-rich byproduct of pyrolysis, has gained attention for its ability to improve soil properties and mitigate heavy metal toxicity, enhancing plant resilience. This study examines biochar’s role in alleviating Cd stress in forage soybean (*Glycine max* L. Merrill), focusing on its effects on growth, nutrient uptake, antioxidant enzyme activities, and phytohormone regulation. A greenhouse experiment was conducted using two biochar levels (B0: Control, B1: 3% biochar) and four Cd concentrations (Cd0: Control, Cd1: 50 mg kg^-1^, Cd2: 100 mg kg^-1^ and Cd3: 200 mg kg^-1^) in a 2 × 4 factorial design with three replications. Biochar and Cd were applied to mixtures consisting of soil (loamy), sand, and peat (3:1:1, v:v:v). Growth parameters, mineral content, antioxidant enzyme activities (catalase-CAT, peroxidase-POD and superoxide dismutase-SOD), and stress indicators (hydrogen peroxide H_2_O_2_, malondialdehyde-MDA, proline and sucrose) were analyzed. Key phytohormones, including abscisic acid-ABA, indole acetic acid-IAA, gibberellic acid-GA, salicylic acid-SA, cytokinin and jasmonic acid-JA, were measured. Cd stress reduced plant growth and nutrient content while increasing oxidative stress markers and antioxidant enzyme activity. Compared to the B0Cd0 treatment, the B0Cd1, B0Cd2, and B0Cd3 treatments resulted in 45, 62 and 67% decrease in shoot fresh weight (SFW), 37, 46 and 50% decrease in shoot dry weight (SDW), 31, 45 and 56% in root fresh weight (RFW), 34, 50 and 59% in root dry weight (RDW), and 13, 29 and 40% de-crease in LA. Biochar mitigated these negative effects by enhancing growth, nutrient assimilation, and biochemical responses. Under Cd stress, biochar reduced H_2_O_2_, MDA, proline and sucrose accumulation, and modulated enzymatic activities, improving stress tolerance in soybean plants. Based on these findings, it is thought that hazelnut shell biochar can effectively alleviate Cd-induced stress in forage soybean by promoting growth, nutrient uptake, and biochemical stability. However, further studies are recommended to explore the use of hazelnut shell biochar as a sustainable soil amendment for reducing heavy metal toxicity in agricultural systems.

## 1 Introduction

The growing global economy, technological progresses and industrial activities have led to enhanced wastes containing heavy metals and many other pollutions (Fattahi et al., 2021). Many parts of the world have soils exposed to heavy metal contamination. The sources of these heavy metals are reported mainly to be anthropogenic activities such as mining (Li et al., 2014), exhaust emissions, wastewater irrigation (Zhang et al., 2008), sewage sludge applications and the use of chemical fertilizers (Yong et al., 2014; Hatamian et al., 2018; Zivdar et al., 2016; Souri et al., 2016). Heavy metals (As, Al, Cd, Hg, Pb, etc.) can exhibit phytotoxic effects on plants and disrupt vital processes in the plant nutrient cycle (Oliveira et al., 2005). One of these heavy metals, Cd, can originate from parent materials as well as from the use of phosphate fertilizers and sewage sludge, in addition to the accumulation of polluted air and industrial wastes in the soil (Huang et al., 2022; Ankush et al., 2024). Cadmium can be easily taken up by roots from the soil and accumulate in branches, stems, and leaves, negatively affecting the development of generative and vegetative organs. Growth retardation or inhibition and sometimes leaf chlorosis are common symptoms of Cd toxicity in plants (Hatamian et al., 2019; Souri and Hatamian, 2019). There are important phosphate reserves in Turkey. 70-80% of the Cd found in raw phosphate rocks used in phosphorus fertilizer production passes into the products used in fertilizer production. With the use of these products, Cd reaches the soil directly through phosphorus-containing fertilizers. Studies have been conducted to rehabilitate soils contaminated with heavy metals for sustainable agriculture (Yurdakul, 2015). These studies have highlighted that remediation techniques are often expensive and labor-intensive. As an alternative, the low-cost and environmentally friendly phytoremediation technique, which uses hyper-accumulator plants capable of accumulating heavy metals in their above-ground organs at rates ranging from 50 to 500 times, has gained prominence (Shi et al., 2009). For this purpose, the phytoremediation potential of plants such as *Alphin penngrass*, Mouse-ear thale-cress, Chinese mustard, garden tomato, corn, barley, rice, oat as well as soybean (*Glycine max* L. Merrill), which is an excellent source of oil and protein for both humans and animals (Wang et al., 2015), has been evaluated (Li et al., 2018; Schwalbert et al., 2019).

Forage crop cultivation has been ongoing since the past because livestock is an important source of livelihood in Turkey. Soybeans used for animal feed are also imported in significant quantities, with soybean meal being the primary import (Tan and Yolcu, 2021). Studies have indicated that soybean plants can be used for remediation in soils contaminated with heavy metals as Cd (Liu et al., 2022). However, it has also been reported that the yield and quality of soybean, which has a wide cultivation area, particularly in agricultural lands near industrial zones, significantly decrease in soils contaminated with heavy metals, especially Cd (Vollmann et al., 2015). While certain agronomic practices have been identified to mitigate the impact of Cd-contaminated soils on soybean plant (Wang et al., 2019), this has highlighted the potential of biochar, a material widely utilized today, for this specific application.

Biochar is a porous, low-density, carbon-rich product formed by the pyrolysis of plant or animal-based biomass at high temperatures in environments with little to no oxygen to low-oxygen conditions.

(Ahmad et al., 2014). Biochar can enhance plant production due to its properties such as resistance to degradation, negative surface charge, and high surface area, while also improving the biological, physical, and chemical properties of soils (Madari et al., 2017; Zhang et al., 2017). Indeed, many researchers have stated that some soil properties can be improved with biochar, and that biochar can regulate soil pH by in-creasing soil pH, thereby inhibiting heavy metal uptake (Budak et al., 2023). Our earlier studies have showed that biochar alleviates the negative effects of salinity (Kul et al., 2021; Gullap et al., 2024a) drought stress (Yildirim et al., 2021, 2022) and Cd stress (Dadasoglu et al., 2022) on plants grown under these conditions, which is particularly effective in 2.5% and 3% doses of biochar.

Biochar is mixed directly into the soil to improve its physical, chemical, and biological properties. Biochar can be added to compost piles to enhance the composting process, reduce nutrient leaching, and create a more stable and nutrient-rich final product. Biochar can be used to immobilize heavy metals, pesticides, and other contaminants in soil, preventing their uptake by crops and leaching into groundwater (Madari et al., 2017; Zhang et al., 2017). Studies have shown that it may have significant potential against a variety of pollutants. Indeed, Zhu et al. (2025) examined how the electronic structure of biochar, oxygen, and peroxydisulfate work together to more efficiently clean polluted water. They found that N-biochar, used against groundwater contaminants, accelerated the degradation of organic pollutants through electron transfer oxidation, increasing degradation efficiency from 5% to 100%. The researchers reported that N-biochar-mediated dissolved oxygen modulation may be effective in improving the treatment of contaminated groundwater.

Various plant wastes, animal manures, cotton and sugarcane waste, and waste wood materials can be used for biochar production, which has significant potential in agricultural waste management (Murtaza et al., 2025). As a sustainable agricultural waste management procedure, hazelnut shells are a good biomass for use as biochar due to their high cellulose, hemicellulose, and lignin content, providing a high specific surface area, porosity, and degree of carbonization (Zhang et al., 2023). Studies have shown significant effects of hazelnut shell biochar in improving the physicochemical structure of various soils (Özenç et al., 2023), increasing P utilization efficiency (Esmaeili et al., 2025), combating drought stress in plants (Gullap et al., 2024b), and Cr stress from heavy metals (Zhang et al., 2023). Producing an estimated 600–650 thousand tons of hazelnuts each year, Turkey is the world’s primary producer. Currently, the hard shells from these nuts are largely discarded, either burned or left to rot. Instead, these shells offer a rich carbon source that can be converted into biochar, significantly improving soil quality and offering an environmentally sound solution (Tarakçıoğlu et al., 2019). Gullap et al. (2024b) reported that biochar originated from hazelnut shells is rich in terms of humic substances (humic acid + fulvic acid), and its amendment into soil can enhance soybean growth by modulating the plant physiology and biochemistry under drought stress. Despite the widespread use of both plant-based materials (such as woody debris, garden waste, and agricultural harvest residues) and non-plant-based materials (including animal manures and waste activated sludge) for biochar production, research on biochar derived from hazelnut shells remains limited. This is notable considering that hazelnut shells are an environmental waste generated in significant volumes with limited applications beyond fuel. Therefore, this study aims to investigate the physiological and biochemical properties of soybean plants grown in Cd-contaminated soils amended with biochar based on hazelnut shell. This research represents a novel investigation into the role of hazelnut shell biochar in mitigating cadmium toxicity in feed soybean.

## 2 Materials and methods

### 2.1 Plant material, growth media and experiment design

The study was conducted under controlled greenhouse conditions (14-hour light/10-hour dark, average temperature 25 ± 2.5°C, relative humidity 54 ± 3.2%) in 2022. The soybean (*Glycine max* L. Merrill) variety “Yeşilsoy,” registered by the Eastern Mediterranean Agricultural Research Institute and suitable for silage, was used as the material in the study.

In the study, hazelnut shells obtained from Turkey’s Black Sea region in 2022 April-August were pyrolyzed at 500°C and ground to 2 mm in size for use as biochar. In the experiment, biochar we utilized was derived from hazelnut shells through a thermal conversion process. This conversion process consists of three distinct stages: depolymerization, hydrolysis, and cracking. Production time was <1 h/batch (up to 500°C at 10°C min^-1^) (Kul et al., 2021). The chemical composition of the hazelnut shell biochar utilized in the experiment is as follows: 7.75 pH, 101.73 cmol kg^-1^ CEC, 200 µS m^-1^ EC, 1.59% volatile matter, 2.22% moisture, 80.5% fixed carbon, 2.14% ash, 5.41% humic matter, 1.34% hydrogen, 2.25% oxygen, 1.25% nitrogen, and a calorific value of 7751–7955 kcal kg^-1^. Biochar fractions have low bulk density and available water content, proper aeration capacity, high organic matter content. The bulk density, maximum water holding capacity, aeration capacity, available water content and organic matter content were 0.33 g cm^-3^, 50.24%, 29.54%, 7.01% and 94.24%, respectively (Gullap et al., 2024b). The specific surface area and pore volume were as 71.84 m^2^ mg^-1^ and 0.0095 m^3^ g^−1^, evaluated by Brunauer–Emmett–Teller (BET) equation. Higher specific surface area and porosity are important factors in determining biochar properties. Research has shown that a larger surface area gives biochar more places to adsorb substances (Li et al., 2017). The exact physicochemical mechanisms cannot be definitively confirmed because advanced characterization data (SEM, FTIR, etc.) were not obtained.

In the study, biochar was applied to mixtures consisting of soil, sand, and peat (3:1:1, v:v:v) in containers measuring 720 x 195x25 mm (35 L). For heavy metal stress treatments, cadmium (CdSO_4_.8H_2_O) was mixed with the medium at three different concentrations (0, 50, 100, and 200 mg kg^-1^) and incubated for 3 weeks. The biochar dose used in the trial was chosen based on the results obtained from our previous research (Kul et al., 2021; Yildirim et al., 2021, 2022; Dadasoglu et al., 2022; Gullap et al., 2024a). The experiment was designed with two biochar levels (B0: Control, B1: 3% biochar) and four Cd levels (Cd0: Control, Cd1: 50 mg kg^-1^, Cd2: 100 mg kg^-1^, and Cd3: 200 mg kg^-1^), resulting in eight different combinations (T_0_: B0Cd0, T_1_: B0Cd1, T_2_: B0Cd2, T_3_: B0Cd3, T_4_: B1Cd0, T_5_: B1Cd1, T_6_: B1Cd2 and T_7_: B1Cd3) arranged in a 2 x 4 factorial design with three replications. In the study, 30 soybean seeds were sown in each pot. Then after germination 20 plants left in two rows. The pots were randomly placed on the benches. There was a 50 cm space between the pots and they were rotated every two days to avoid positional effects. Soil moisture levels were adjusted by using a moisture meter (HH2 Moisture Meter, Delta-T Devices, Cambridge, England). The Pots were irrigated by maintaining field capacity at each irrigation.

### 2.2 Plant harvest and analysis

In the conducted study, soybean seedlings, harvested 45 days after sowing, were subjected to comprehensive analyses to evaluate their morphological, physiological, and biochemical characteristics. For all analyses 10 plants selected randomly. The study assessed a number of parameters including shoot fresh weight (SFW), root fresh weight (RFW), shoot dry weight (SDW), root dry weight (RDW), leaf area (LA), chlorophyll content (chlorophyll-a, chlorophyll-b, and total chlorophyll), and electrical leakage (EL). Essential mineral nutrients such as nitrogen (N), phosphorus (P), potassium (K), calcium (Ca), magnesium (Mg), sulfur (S), manganese (Mn), iron (Fe), zinc (Zn), boron (B), sodium (Na), and Cd were also quantified. Additionally, the activities of antioxidant enzymes such as superoxide dismutase (SOD), peroxidase (POD), and catalase (CAT) were measured, along with the concentrations of growth regulators like indole-3-acetic acid (IAA), abscisic acid (ABA), gibberellic acid (GA), salicylic acid (SA), cytokinin-CK (zeatin and zeatin riboside, Z + ZR), zeatin, and jasmonic acid. To evaluate the biochemical and physiological responses of the soybean seedlings, the levels of hydrogen peroxide (H_2_O_2_), malondialdehyde (MDA), proline, and sucrose were analyzed.

Upon harvesting the plants at soil level, the fresh weights of the shoots and roots were immediately measured. Fresh leaf samples were collected and stored at -80°C to preserve their structural and biochemical properties for subsequent analyses. The plant materials were then oven-dried at 67°C for 48 hours to determine the dry weights of the shoots and roots.

### 2.3 Leaf area and assay of chlorophyll

Leaf area was measured using a CI-202 Portable Area Meter (CID, Inc., USA), and chlorophyll content, including chlorophyll a, chlorophyll b, and total chlorophyll, was analyzed following the method of Lichtenthaler and Wellburm (1983).

### 2.4 Mineral analysis

The mineral content of the leaves was determined using an Optima 2100 DV ICP/OES spectrophotometer (Perkin-Elmer, Shelton, CT), as described by Mertens (2005a) and Mertens (2005b). Nitrogen content was quantified using the Micro Kjeldahl wet combustion method of Bremner (1996). The soil used in the study has a loamy texture, a neutral pH of 7.45, a very low organic matter content of 0.93%, a low electrical conductivity of 116.5 m^S^ m^-1^ indicating low salinity, and a lime (CaCO_3_) content of 2.68%, classifying it as calcareous. The soil’s NH_4_+N, NO_3_-N, P, B, Cu, Fe, Zn, and Mn contents were as 3.33, 1.81, 4.15, 0.05, 0.95, 6.36, 0.16 and 0.26 mg kg^-1^, respectively. Additionally, K^+^, Ca^2+^, Mg^2+^ and Na^+^ contents were as 0.6, 8.33, 0.83 and 0.09 mg kg^-1^, respectively (Mertens, 2005b; Bremner, 1996). A baseline measurement of DTPA-extractable cadmium was not conducted (Sahu et al., 2022).

### 2.5 Lipid peroxidation (measurement of malondialdehyde-MDA) and hydrogen peroxide (H_2_O_2_)

Lipid peroxidation was quantified by measuring the content of malondialdehyde (MDA). A 0.2 g sample of frozen leaves was finely ground in liquid nitrogen and extracted with 3 ml of cold ethanol. After centrifuging the crude extract at 12,000×g for 20 min, the supernatant was mixed with trichloroacetic acid (TCA), thiobarbituric acid (TBA), and butylated hydroxytoluene (BHT). This mixture was heated to promote the reaction, rapidly cooled in an ice bath to stop it, and then centrifuged again. The absorbance of the final supernatant, representing the TBA-reactive substances (TBARS) with MDA as the lipid degradation product, was measured at 400, 500, and 600 nm. The MDA concentration was calculated using an extinction coefficient of 155 mmol L^–1^ cm^−1^ (Shams et al., 2019).

The hydrogen peroxide (H_2_​O_2_​) content was measured using the method described by Velikova et al. (2000). H_2_​O_2_​ was extracted by homogenizing 200 mg of leaf tissue in 2 ml of 0.1% (w/v) trichloroacetic acid (TCA) on ice, followed by centrifugation at 12,000×g for 15 min. A 0.4 ml aliquot of the supernatant was then combined with 0.4 ml of 10 mmol L^−1^ potassium phosphate buffer (pH 7.0) and 0.8 ml of 1 mol L^−1^ potassium iodide (KI). The final mixture’s absorbance was read at 390 nm. The H_2_​O_2_​ concentration was determined by referencing a previously constructed standard calibration curve (Shame et al., 2019).

### 2.6 Assay of antioxidant enzyme activity

To analyze antioxidant enzymes, frozen leaves were finely ground using liquid nitrogen. The resulting powder was then extracted with an ice-cold solution consisting of 0.1 M Tris-HCl buffer (pH 7.5), 5% (w/v) sucrose, and 0.1% 2-mercaptoethanol (a 3:1 buffer volume-to-fresh weight ratio). Next, the homogenate was centrifuged at 10,000 g for 20 minutes at 4°C. Finally, the supernatant was collected for enzyme activity measurements (Havir and Mchale, 1987; Angelini et al., 1990; Yordanova et al., 2004). All preparation steps for extraction and assay were performed at 4°C. CAT activity was quantified by monitoring the decrease in H_2_O_2_ at 240 nm in a reaction mix containing 50 mM phosphate buffer (pH 7.0), 10 mM H_2_O_2_, and 100-μL extract, using an extinction coefficient of 39.4 mM ^−1^ cm ^−1^ (Abedi and Pakniyat, 2010). The activity was measured in a reaction mixture (3.0 ml final volume) composed of 30 mM H_2_O_2_ in 50 mM NaKPi, pH 7.0, and 2.0 ml of enzyme extract. Samples without H_2_O_2_ were used as a blank. The decomposition of H_2_O_2_ was followed. Peroxidase (POD) activity was measured using the method described by Abedi and Pakniyat (2010). The assay quantified the enzyme’s ability to convert guaiacol to tetraguaiacol, monitored as an increase in absorbance at 436 nm. The molar extinction coefficient (∈) for tetraguaiacol was utilized as 26.6 mM^−1^ cm^−1^. The complete reaction mixture consisted of 100 mM K-phosphate buffer (pH 7.0), 20.1 mM guaiacol, 10 mM H_2_O_2_, and the necessary enzyme extract. The reaction was initiated by adding H_2_O_2_, and the resulting increase in absorbance was recorded at 436 nm for 5 minutes. The Superoxide Dismutase (SOD) activity was assessed following the method of Abedi and Pakniyat (2010), which utilizes the enzyme’s capacity to inhibit the light-dependent reduction of nitroblue tetrazolium (NBT), monitored at 560 nm. The full reaction volume was composed of 50 mM K-phosphate buffer (pH 7.8), 13 mM methionine, 75μM NBT, 0.1μM EDTA, 4μM riboflavin (the initiator), and the enzyme extract. Riboflavin addition started the reaction, followed by 15 minutes of exposure to two 15 W fluorescent lights. A sample without enzyme served as the maximal color control, and a non-irradiated sample acted as the blank. SOD activity was quantified, with one unit being the enzyme amount that caused a 50% decrease in NBT reduction, measured spectrophotometrically.

### 2.7 Measurement of electrolyte leakage

For the measurement of electrolyte leakage (EL), 10 leaf discs (10 mm in diameter) from the young fully expanded leaves from two plants per replicate were placed in 50-mL glass vials and rinsed with distilled water to remove the electrolytes released during leaf disc excision. Vials were then filled with 30 mL of distilled water and allowed to stand in the dark for 24 h at room temperature. Electrical conductivity (EC1) of the bathing solution was determined at the end of the incubation period. Vials were heated in a temperature-controlled water bath at 95 °C for 20 min and then cooled to room temperature and the (EC2) was again measured. Electrolyte leakage was calculated as a percentage of EC1/EC2.

### 2.8 Leaf relative water content

Leaf relative water content: Two leaves were collected among the young fully expanded leaves of two plants per replicate. Individual leaves detached from the stem were weighed to determine fresh weight (FW). To determine the turgid weight (TW), leaves kept floating in distilled water inside a closed Petri dish. Leaf samples were weighed periodically, after gently wiping the water from the surface with the tissue paper until a steady weight was achieved. At the end of imbibition period, leaf samples were placed in a preheated oven at 70°C for 48 h to determine dry weight (DW). Values of FW, TW and DW were used to calculate leaf relative water content (LRWC) using the following equation: LRWC (%) = [(FW−DW)/(TW−DW)]×100.

### 2.9 Assay of phytohormone

To extract and purify phytohormones, researchers followed the protocol by Kuraishi et al. (1991) and Koshita et al. (1999). One gram of fresh leaf sample was homogenized in 80% methanol at –40°C for 10 minutes, then incubated in the dark for 24 hours. After the samples were dried at 35 °C using an evaporator, the residue was redissolved in 0.1M KHPO_4_ (pH 8.0) and purified with a Sep-Pak C-18 cartridge. The hormones were then eluted with 80% methanol. For analysis, the purified hormones were run on a Zorbax Eclipse-AAA C-18 column (Agilent 1200 HPLC) using a mobile phase of 13% acetonitrile at pH 4.98. The analysis was conducted at a flow rate of 1.2 mL min^–1^ and a column temperature of 25°C. A UV detector at 265 nm was used to identify gibberellic acid (GA3), salicylic acid (SA), indole acetic acid (IAA), and abscisic acid (ABA), as described by Turan et al. (2014). Standard curves were made using ABA [(±)-ABA, A1049], GA3 (G7645), SA (S7401), and IAA (I2886). QA/QC for phytohormones evaluations referred to the method reported in a previous study (Shi et al., 2015).

### 2.10 Assay of proline

A 50 mg of frozen leaf sample was powdered with liquid nitrogen and extracted with a pestle and mortar with 4.5 ml of 5-sulfosalicylic acid 3% in an ice bath. The homogenates were filtered with a filter paper (#2). Two ml of filtrate was reacted with 2 ml acid-ninhdrin and 2 ml of glacial acetic acid in a test tube for 1 h at 100°C, and the reaction terminated in an ice bath. The filtrates were used for the analysis. Proline concentration was assayed spectrophotometrically at 520 nm (Bates et al., 1973; Chopra et al., 2000).

### 2.11 Assay of sucrose

Sucrose concentration was determined using the method of Liu and Huang (2000). First, 0.1 g of the dry sample was extracted by incubating it in 10 ml of 0.1 M phosphate buffer (pH 5.4) for 24 h at 22°C. To measure sucrose, a 0.2 ml aliquot of the resulting supernatant was mixed with distilled water and 1.0 ml of invertase (10 U ml^−1^) and then incubated in a water bath for 1 h at 50°C. Following the principle described by Ting (1956), the final sucrose content was calculated based on the difference in the reduction of total sugar content between the reaction mixture containing invertase and a control mixture without the enzyme.

### 2.12 Statistical analysis

The study was established according to the randomized plots experimental design with three replications and 6 pots in each replication. The experimental design was hierarchical with respect to two factors arranged in a completely randomized design with four replications. The first factor (Cd levels) had four levels (Cd0: Control, Cd1: 50 mg kg^-1^, Cd2: 100 mg kg^-1^, and Cd3: 200 mg kg^-1^), and the second one (biochar treatments) had two levels ((B0: Control, B1: 3% biochar). The data were subjected to a two-way ANOVA in a 2 (biochar) x 4 (cadmium) factorial design according to a completely random design. Differences between means were determined with Tukey’s Honestly Significant Difference (HSD) *post hoc* test at 0.05 level. All statistical analyses were performed using SPSS 20.0 statistical package. Graphs were prepared using Excel 2019 from Microsoft Corporation, USA.

## 3 Results

The findings of the study pointed out that the increasing Cd levels had a negative impact on the growth characteristics of soybean seedlings under normal conditions (**Table 1**). Compared to the T_0_ treatment, the T_1_, T_2_, and T_3_ treatments resulted in 45, 62 and 67% decrease in SFW, 37, 46 and 50% decrease in SDW, 31, 45 and 56% in RFW, 34, 50 and 59% in RDW, and 13, 29 and 40% decrease in LA. The negative effects of Cd on soybean growth parameters were generally mitigated by the biochar used in the study (**Table 1**).

**Table 1:** Effect of hazelnut shell biochar on the growth characteristics of forage soybean in Cd-contaminated soils (***: p<0.001).

Treatments	SFW (g)	SDW (g)	RFW (g)	RDW (g)	LA (cm^2^ plant^-1^)
T_0_	13.47 ± 0.33 c	2.35 ± 0.14 b	1.78 ± 0.08 d	0.32 ± 0.02 d	239.74 ± 5.58 ab
T_1_	7.45 ± 0.18 e	1.49 ± 0.06 c	1.22 ± 0.04 e	0.21 ± 0.01 e	208.89 ± 4.11 c
T_2_	5.07 ± 0.23 f	1.28 ± 0.03 d	0.98 ± 0.05 fg	0.16 ± 0.01 f	169.86 ± 7.70 d
T_3_	4.38 ± 0.19 f	1.17 ± 0.04 d	0.78 ± 0.03 g	0.13 ± 0.03 f	142.80 ± 11.46 e
T_4_	15.74 ± 0.20 a	2.76 ± 0.08 a	2.96 ± 0.05 a	0.56 ± 0.02 a	261.32 ± 1.91 a
T_5_	14.23 ± 0.36 b	2.46 ± 0.05 b	2.26 ± 0.18 b	0.48 ± 0.01 b	225.25 ± 4.25 bc
T_6_	14.42 ± 0.27 b	2.29 ± 0.02 b	2.01 ± 0.04 c	0.39 ± 0.02 c	215.29 ± 3.58 c
T_7_	9.52 ± 0.34 d	1.60 ± 0.04 c	1.07 ± 0.04 ef	0.25 ± 0.01 e	215.73 ± 14.12c
*Mean square*	*61.174*	*1.099*	*1.703*	*0.072*	*4259.800*
*F value*	877.150^***^	254.128^***^	272.674^***^	291.988^***^	72.086^***^
*Error*	*0.070*	*0.006*	*0.004*	*0.000*	*59.093*

The values are the means of three replicates with standard deviation (± SD). Different letters indicate within each column significant differences at p <0.05 (Tukey HSD test). SFW, Shoot fresh weight; SDW, Shoot dry weight; RFW, Root fresh weight; RDW, Root dry weight; LA, Leaf area. T_0_: B0Cd0, T_1_: B0Cd1, T_2_: B0Cd2, T_3_: B0Cd3, T_4_: B1Cd0, T_5_: B1Cd1, T_6_: B1Cd2 and T_7_: B1Cd3. B0: No biochar, B1: 3% biochar, Cd0: No cadmium, Cd1: 50 mg kg^-1^, Cd2: 100 mg kg^-1^ and Cd3: 200 mg kg^-1^.

Statistical analysis results are italicized to distinguish them from average data.

As the Cd concentration increased, a decrease in chlorophyll content was observed, while an increase in electrical leakage (EL) was recorded (**Table 2**). In terms of chlorophyll-a values, compared to the T_0_ treatment, the T_1_, T_2_, and T_3_ treatments showed reductions of approximately 13%, 23%, and 39%, respectively. The highest chlorophyll-a content was recorded in the T_4_ (B1Cd0) treatment, while the lowest content was observed in the treatment without biochar and with 200 mg kg^-1^ Cd (T_3_: B0Cd3). In the study, chlorophyll-b content decreased by approximately 30%, 28%, and 54% in the T_1_, T_2_, and T_3_ treatments, respectively, compared to the T_0_ treatment. Similarly, compared to the T_4_ treatment, chlorophyll-b content decreased by approximately 9%, 15%, and 29% in the T_5_, T_6_, and T_7_ treatments, respectively (**Table 2**). The lowest chlorophyll-b content was detected in the T_3_ (B0Cd3) treatment, while the highest chlorophyll-b content was recorded in the T_4_ (B1Cd0) treatment. The total chlorophyll content of forage soybean seedlings also showed significant decreases with increasing Cd levels, similar to the trends observed for chlorophyll-a and chlorophyll-b. Compared to the T_0_ treatment, the average total chlorophyll content decreased by 19, 25 and 44% in the T_1_, T_2_, and T_3_ treatments, while it decreased by 5, 10 and 19% in the T_5_, T_6_, and T_7_ treatments compared to the T_4_ treatment (**Table 2**). When examining **Table 2**, it was observed that the EL values increased by 15, 56 and 106% in the T1, T_2_, and T_3_ treatments compared to the T_0_ treatment. Similarly, the EL values increased by 21, 22 and 49% in the T_5_, T_6_, and T_7_ treatments com-pared to the T_4_ treatment.

**Table 2:** Effect of hazelnut shell biochar on chlorophyll content and electrical leakage of forage soybean in Cd-contaminated soils (***: p<0.001).

Treatments	Chl-a (mg g^-1^)	Chl-b (mg g^-1^)	Total Chl (mg g^-1^)	EL (%)
T_0_	2.76 ± 0.05 a	1.55 ± 0.05 b	4.31 ± 0.10 ab	31.40 ± 1.67 de
T_1_	2.39 ± 0.10 c	1.09 ± 0.02 d	3.48 ± 0.12 d	36.23 ± 1.83 d
T_2_	2.12 ± 0.02 d	1.11 ± 0.04 d	3.23 ± 0.05 e	48.98 ± 3.70 b
T_3_	1.69 ± 0.05 e	0.72 ± 0.04 e	2.41 ± 0.09 f	64.53 ± 1.70 a
T_4_	2.80 ± 0.01 a	1.65 ± 0.02 a	4.45 ± 0.01 a	28.69 ± 1.98 e
T_5_	2.71 ± 0.03 ab	1.50 ± 0.03 b	4.22 ± 0.05 bc	34.81 ± 0.27 d
T_6_	2.62 ± 0.03 b	1.40 ± 0.01 c	4.02 ± 0.04 c	34.95 ± 0.96 d
T_7_	2.43 ± 0.04 c	1.17 ± 0.04 d	3.60 ± 0.06 d	42.83 ± 1.68 c
*Mean square*	*0.429*	*0.287*	*1.393*	*410.522*
*F value*	194.899^***^	231.132^***^	259.557^***^	107.601^***^
*Error*	*0.002*	*0.001*	*0.005*	*3.815*

The values are the means of three replicates with standard deviation (± SD). Different letters indicate within each column significant differences at p <0.05 (Tukey HSD test). Chl-a, Chlorophyll a; Chl-b, Chlorophyll b; Total Chl, Total chlorophyll a; EL, Electrical leakage. T_0_: B0Cd0, T_1_: B0Cd1, T_2_: B0Cd2, T_3_: B0Cd3, T_4_: B1Cd0, T_5_: B1Cd1, T_6_: B1Cd2 and T_7_: B1Cd3. B0: No biochar, B1: 3% biochar, Cd0: No cadmium, Cd1: 50 mg kg^-1^, Cd2: 100 mg kg^-1^ and Cd3: 200 mg kg^-1^.

Statistical analysis results are italicized to distinguish them from average data.

In the study, the effects of biochar on the N, P, K, Ca, Mg, and S content of silage soy-bean in Cd-contaminated soils are presented in **Table 3**, while the effects on Mn, Fe, Zn, B, Na, and Cd content are shown in **Table 4**. The N, P, K, Ca, Mg, and S content of forage soybean decreased with increasing Cd levels (**Table 3**). In the absence of biochar, under Cd stress, the N, P, K, Ca, Mg, and S content of forage soybean seedlings decreased significantly by approximately between 41-63%, 58-70%, 40-48%, 37-51%, 33-57%, and 29-36%, respectively. With B1Cd0 application, N, P, K, Ca, Mg, and S contents under both normal and Cd stress conditions were higher than those in B0Cd0 application (**Table 3**). In the experiment, the highest N, P, K, Ca, Mg, and S content was obtained in the T_4_ (B1Cd0) treatment, while the lowest N, P, K, Ca, and Mg were observed in the T_3_ (B0Cd3) treatment. In conclusion, the study found that biochar applications significantly increased the N, P, K, Ca, Mg, and S content in soybean seedlings compared to the controls (without biochar) (**Table 3**).

**Table 3:** Effect of hazelnut shell biochar on N, P, K, Ca, Mg and S content of forage soybean in Cd-contaminated soils (***: p<0.001).

Treatments	N (%)	P (%)	K (%)	Ca (%)	Mg (%)	S (%)
T_0_	2.95 ± 0.021 b	0.33 ± 0.009 c	1.82 ± 0.037 d	1.15 ± 0.039 c	0.21 ± 0.016 b	0.14 ± 0.01 bc
T_1_	1.23 ± 0.013 e	0.14 ± 0.01 f	1.10 ± 0.008 e	0.73 ± 0.03 d	0.14 ± 0.019 cd	0.15 ± 0.025 bc
T_2_	1.12 ± 0.044 e	0.11 ± 0.008 fg	0.95 ± 0.058 f	0.64 ± 0.029 e	0.11 ± 0.005 de	0.10 ± 0.001 de
T_3_	1.07 ± 0.009 e	0.10 ± 0.003 g	0.95 ± 0.064 f	0.56 ± 0.04 e	0.09 ± 0.01 e	0.09 ± 0.007 de
T_4_	3.15 ± 0.041 a	0.42 ± 0.01 a	2.47 ± 0.036 a	1.42 ± 0.006 a	0.35 ± 0.015 a	0.23 ± 0.021 a
T_5_	2.89 ± 0.119 b	0.38 ± 0.006 b	2.28 ± 0.056 b	1.32 ± 0.023 b	0.32 ± 0.025 a	0.17 ± 0.007 b
T_6_	2.67 ± 0.107 c	0.29 ± 0.009 d	2.25 ± 0.015 b	1.29 ± 0.04 b	0.18 ± 0.01 bc	0.13 ± 0.006 cd
T_7_	2.45 ± 0.061 d	0.23 ± 0.02 e	2.04 ± 0.094 c	1.29 ± 0.01 b	0.13 ± 0.006 de	0.08 ± 0.023 e
*Mean square*	*2.399*	*0.049*	*1.225*	*0.366*	*0.029*	*0.007*
*F value*	569.133^***^	438.934^***^	438.243^***^	407.247^***^	136.867^***^	32.501^***^
*Error*	*0.004*	*0.000*	*0.003*	*0.001*	*0.000*	*0.000*

The values are the means of three replicates with standard deviation (± SD). Different letters indicate within each column significant differences at p <0.05 (Tukey HSD test). T_0_: B0Cd0, T_1_: B0Cd1, T_2_: B0Cd2, T_3_: B0Cd3, T_4_: B1Cd0, T_5_: B1Cd1, T_6_: B1Cd2 and T_7_: B1Cd3. B0: No biochar, B1: 3% biochar, Cd0: No cadmium, Cd1: 50 mg kg^-1^, Cd2: 100 mg kg^-1^ and Cd3: 200 mg kg^-1^.

Statistical analysis results are italicized to distinguish them from average data.

**Table 4:** Effect of hazelnut shell biochar on Mn, Fe, Zn, B, Na and Cd content of forage soybean in Cd-contaminated soils (***: p<0.001).

Treatments	Mn (mg kg^-1^)	Fe (mg kg^-1^)	Zn (mg kg^-1^)	B (mg kg^-1^)	Na (mg kg^-1^)	Cd (mg kg^-1^)
T_0_	22.02 ± 2.18 c	49.8 ± 2.06 c	12.86 ± 1.09 bc	4.94 ± 0.39 a	6.11 ± 0.39 b	0.09 ± 0.006 e
T_1_	17.19 ± 1.95 d	40.04 ± 2.14 d	7.58 ± 0.40 de	4.49 ± 0.13 a	6.67 ± 0.60 b	2.58 ± 0.178 c
T_2_	12.96 ± 0.60 e	28.71 ± 1.79 e	5.62 ± 0.19 e	2.49 ± 0.10 bc	3.75 ± 0.11 cd	4.66 ± 0.125 b
T_3_	9.34 ± 0.98 e	23.53 ± 1.64 e	5.07 ± 0.25 e	1.83 ± 0.32 c	3.73 ± 0.07 cd	7.57 ± 0.585 a
T_4_	35.99 ± 2.35 a	82.09 ± 2.24 a	22.01 ± 2.74 a	3.59 ± 0.22 ab	8.86 ± 0.43 a	0.03 ± 0.002 e
T_5_	26.26 ± 1.04 b	76.31 ± 2.23 a	16.79 ± 2.06 b	2.34 ± 0.31 bc	3.24 ± 0.14 d	1.49 ± 0.103 d
T_6_	20.60 ± 0.92 cd	65.74 ± 4.74 b	13.15 ± 1.73 bc	1.41 ± 0.35 c	5.48 ± 0.29 bc	2.68 ± 0.072 c
T_7_	20.65 ± 0.32 cd	56.94 ± 3.46 c	11.57 ± 2.40 cd	1.31 ± 0.30 c	3.90 ± 0.34 cd	4.35 ± 0.337 b
*Mean square*	*200.576*	*1373.197*	*100.021*	*5.767*	*11.255*	*19.419*
*F value*	92.046^***^	185.638^***^	36.597^***^	20.940^***^	28.385^***^	299.675^***^
*Error*	*2.179*	*7.397*	*2.733*	*0.275*	*0.397*	*0.065*

The values are the means of three replicates with standard deviation (± SD). Different letters indicate within each column significant differences at p <0.05 (Tukey HSD test). T_0_: B0Cd0, T_1_: B0Cd1, T_2_: B0Cd2, T_3_: B0Cd3, T_4_: B1Cd0, T_5_: B1Cd1, T_6_: B1Cd2 and T_7_: B1Cd3. B0: No biochar, B1: 3% biochar, Cd0: No cadmium, Cd1: 50 mg kg^-1^, Cd2: 100 mg kg^-1^ and Cd3: 200 mg kg^-1^.

Statistical analysis results are italicized to distinguish them from average data.

The Mn, Fe, Zn, B, and Na content of forage soybean decreased with increasing Cd levels, while the Cd content increased. In the study, compared to the T0 treatment, the Mn, Fe, Zn, B, and Na content decreased by an average of 40%, 38%, 53%, 31%, and 29%, respectively, in the T_1_, T_2_, and T_3_ treatments. Similarly, compared to the T_4_ treatment, the Mn, Fe, Zn, B, and Na content decreased by an average of 38%, 19%, 37%, 53%, and 52%, respectively, in the T5, T6, and T7 treatments. However, Mn, Fe, Zn, B, and Na values in T_4_, T_5_, T_6_ and T_7_ applications were higher than in B0 applications (**Table 4**).

The highest Mn, Fe, Zn, and Na contents were recorded in the T_4_ (B1Cd0) treatment, while the highest B content was observed in the T_0_ (B0Cd0) treatment. On the other hand, the lowest Mn, Fe, and Zn contents were detected in the T_3_ (B0Cd3) treatment, the lowest B content in the T_7_ (B1Cd3) treatment, and the lowest Na content in the T_5_ (B1Cd1) treatment. Additionally, the highest Cd content in the experiment was found in the T_3_ (B0Cd3) treatment, followed by the T_2_ (B0Cd2) treatment. The lowest Cd content was determined in the T_4_ (B1Cd0) treatment, followed by the T_0_ (B0Cd0) treatment (**Table 4**).

The effects of biochar on some physiological properties of forage soybean, such as H_2_O_2_, MDA, proline, and sucrose content, in Cd-contaminated soils are presented in **Table 5**. In the study, it was observed that the increase in Cd levels significantly affected all the physiological properties examined in forage soybean, regardless of the biochar dose (**Table 5**). When the control (T_0_) treatment was compared with the T_1_ (B0Cd1) treatment, the H_2_O_2_, MDA, proline, and sucrose content in soybean seedlings increased by 109%, 359%, 130%, and 633%, respectively. On the other hand, when the T_4_ treatment was compared with the T_0_ treatment, the H_2_O_2_ and MDA, content decreased by 11% and 27% while proline and sucrose content in soybean seedlings increased by 20% and 17%. T_5_, T_6_ and T_7_ treatments were compared with the T_0_ treatment, the H_2_O_2_ content increased by 10%, 144% and 267% and MDA content increased by 322%, 425% and 805%, respectively (**Table 5**).

**Table 5:** Effect of hazelnut shell biochar on some physiological properties of forage soybean in Cd-contaminated soils (***: p<0.001).

Treatments	H_2_O_2_ (mmol kg^-1^)	MDA (mmol kg^-1^)	Proline (%)	Sucrose (%)
T_0_	21.86 ± 0.69 f	3.49 ± 0.56 f	0.10 ± 0.002 d	0.48 ± 0.05 e
T_1_	45.68 ± 1.06 e	16.03 ± 0.64 de	0.23 ± 0.021 c	3.52 ± 0.09 bc
T_2_	59.04 ± 1.36 c	20.32 ± 0.57 c	0.34 ± 0.013 b	3.89 ± 0.12 b
T_3_	96.86 ± 1.19 a	40.60 ± 2.88 a	0.43 ± 0.032 a	4.81 ± 0.22 a
T_4_	19.41 ± 1.63 f	2.54 ± 0.40 f	0.12 ± 0.006 d	0.56 ± 0.06 e
T_5_	24.13 ± 1.00 f	14.73 ± 0.56 e	0.20 ± 0.010 c	2.56 ± 0.04 d
T_6_	53.37 ± 3.78 d	18.32 ± 12.36 cd	0.24 ± 0.020 c	2.66 ± 0.02 d
T_7_	80.20 ± 2.59 b	31.60 ± 1.15 b	0.33 ± 0.031 b	3.45 ± 0.11 c
*Mean square*	*2406.845*	*498.717*	*0.038*	*7.135*
*F value*	652.202^***^	324.808^***^	96.043^***^	371.690^***^
*Error*	*3.690*	*1.535*	*0.000*	*0.019*

The values are the means of three replicates with standard deviation (± SD). Different letters indicate within each column significant differences at p <0.05 (Tukey HSD test). H_2_O_2_, Hydrogen peroxide; MDA, Malondialdehyde. T_0_: B0Cd0, T_1_: B0Cd1, T_2_: B0Cd2, T_3_: B0Cd3, T_4_: B1Cd0, T_5_: B1Cd1, T_6_: B1Cd2 and T_7_: B1Cd3. B0: No biochar, B1: 3% biochar, Cd0: No cadmium, Cd1: 50 mg kg^-1^, Cd2: 100 mg kg^-1^ and Cd3: 200 mg kg^-1^.

Statistical analysis results are italicized to distinguish them from average data.

The increase in Cd application significantly elevated the activity of antioxidant enzymes, while biochar application reduced the antioxidant activity in soybean plants un-der Cd stress. The activities of CAT, POD, and SOD enzymes increased by approximately 45%, 15%, and 37%, respectively, in the T_2_ treatment compared to the T_1_ treatment, and by approximately 29%, 29%, and 36%, respectively, in the T_3_ treatment compared to the T_2_ treatment. Similarly, in the biochar application, the activities of CAT, POD, and SOD enzymes increased by approximately 32%, 21%, and 38%, respectively, when the T_5_ treatment was compared to the T_6_ treatment. When the T_6_ treatment was compared to the T_7_ treatment, the activities of CAT and POD enzymes increased by 55% and 4%, respectively, while the activity of the SOD enzyme decreased by 25%. In the study, it was observed that the increase in Cd stress significantly elevated the activity of antioxidant enzymes, while biochar application reduced the antioxidant activity in soybean seedlings under Cd stress. However, the CAT, POD, and SOD enzyme activities reached their highest values in the T_0_, T_1_, T_2_, and T_3_ treatments, while in the hazelnut shell biochar applications, the same enzyme activities were found to have lower values in the T_4_, T_5_, T_6_, and T_7_ treatments (**Table 6**).

**Table 6:** Effect of hazelnut shell biochar on antioxidant enzyme activities of forage soybean in Cd-contaminated soils (***: p<0.001).

Treatments	CAT (eu g^-1^)	POD (eu g^-1^)	SOD (eu g^-1^)
T_0_	69.14 ± 6.33 f	1625.13 ± 36.54 f	43.33 ± 4.16 d
T_1_	511.45 ± 5.79 d	4601.45 ± 117.04 d	688.00 ± 45.90 c
T_2_	741.23 ± 48.90 c	5295.63 ± 91.44 b	944.67 ± 49.97 b
T_3_	959.04 ± 50.78 a	6821.37 ± 67.04 a	1288.00 ± 66.01 a
T_4_	58.33 ± 2.89 f	1477.67 ± 45.35 f	52.33 ± 2.52 d
T_5_	402.45 ± 5.51 e	4080.00 ± 39.05 e	700.00 ± 20.00 c
T_6_	529.21 ± 19.13 d	4933.00 ± 87.50 c	965.67 ± 17.79 b
T_7_	822.41 ± 10.80 b	5141.00 ± 90.02 bc	721.67 ± 22.55 c
*Mean square*	*326993.384*	*10154474.980*	*566855.518*
*F value*	*470.077*	*1719.996*	*444.172*
*Error*	695.616^***^	5903.777^***^	1276.208^***^

The values are the means of three replicates with standard deviation (± SD). Different letters indicate within each column significant differences at p <0.05 (Tukey HSD test). CAT, Catalase; POD, Peroxidase; SOD, Superoxide dismutase. T_0_: B0Cd0, T_1_: B0Cd1, T_2_: B0Cd2, T_3_: B0Cd3, T_4_: B1Cd0, T_5_: B1Cd1, T_6_: B1Cd2 and T_7_: B1Cd3. B0: No biochar, B1: 3% biochar, Cd0: No cadmium, Cd1: 50 mg kg^-1^, Cd2: 100 mg kg^-1^ and Cd3: 200 mg kg^-1^.

Statistical analysis results are italicized to distinguish them from average data.

**Table 7** indicates the impact of biochar application on the levels of growth hormones in forage soybean, including IAA, ABA, GA, SA, CK, zeatin, and jasmonic acid, under Cd-contaminated soil conditions. In the study conducted with forage soybean plants, as Cd stress increased, the content of IAA, GA, SA, CK, zeatin, and JA decreased, while the ABA content increased. However, application of biochar to soybean seedlings under Cd stress resulted in an increase in IAA, GA, SA, CK, zeatin and JA contents and a decrease in ABA content compared to the T0 ap-plication (**Table 7**). In the study, the levels of IAA, GA, SA, CK, zeatin, and jasmonic acid content decreased by an average of 85%, 82%, 84%, 75%, 74%, and 76%, respectively, in the T_1_, T_2_ and T_3_ treatments compared to the T0 treatment. Similarly, compared to the T_0_ treatment, these hormone levels increased by 80%, 28%, 37%, 24%, 40%, and 92%, respectively, in the T_4_ treatment. In the study, it was observed that the increase in ABA content in bio-char applications was lower than in the control applications (**Table 7**).

**Table 7:** Effect of hazelnut shell biochar on the growth hormone content of forage soybean in Cd-contaminated soils (***: p<0.001).

Treatments	IAA ng mg tissue^-1^	ABA ng g DW^-1^	GA ng g DW^-1^	SA ng g DW^-1^	CK (Z + ZR) ng g DW^-1^	Zeatin ng g DW^-1^	JA ng g DW^-1^
T_0_	1.43 ± 0.07 b	263.25 ± 30.91 f	2.01 ± 0.01 b	0.41 ± 0.012 b	4.24 ± 0.09 b	1.42 ± 0.05 b	0.26 ± 0.019 c
T_1_	0.32 ± 0.08 de	3453.50 ± 66.64 d	0.46 ± 0.04 f	0.10 ± 0.007 d	1.47 ± 0.01 f	0.47 ± 0.04 d	0.08 ± 0.004 d
T_2_	0.19 ± 0.01 ef	4326.33 ± 80.70 b	0.32 ± 0.01 g	0.06 ± 0.004 de	0.93 ± 0.06 g	0.40 ± 0.02 d	0.07 ± 0.010 d
T_3_	0.14 ± 0.01 f	6673.33 ± 184.52 a	0.26 ± 0.02 g	0.04 ± 0.001 e	0.79 ± 0.05 g	0.24 ± 0.03 e	0.04 ± 0.004 d
T_4_	2.58 ± 0.05 a	222.33 ± 12.50 f	2.58 ± 0.03 a	0.56 ± 0.037 a	5.27 ± 0.25 a	1.99 ± 0.09 a	0.50 ± 0.023 a
T_5_	0.50 ± 0.05 c	3003.33 ± 36.17 e	1.45 ± 0.06 c	0.39 ± 0.006 b	3.75 ± 0.31 c	1.44 ± 0.06 b	0.34 ± 0.028 b
T_6_	0.35 ± 0.02 d	3940.00 ± 69.28 c	1.07 ± 0.04 e	0.28 ± 0.009 c	3.22 ± 0.06 d	1.38 ± 0.06 bc	0.08 ± 0.003 d
T_7_	0.23 ± 0.02 def	4283.33 ± 35.12 b	1.24 ± 0.02 d	0.25 ± 0.016 c	2.38 ± 0.11 e	1.25 ± 0.05 c	0.06 ± 0.006 d
*Mean square*	*2.213*	*13974800.000*	*2.071*	*0.105*	*8.030*	*1.169*	*0.087*
*F value*	1090.000^***^	2091.421^***^	1802.947^***^	424.968^***^	338.643^***^	446.428^***^	376.386^***^
*Error*	*0.002*	*6681.965*	*0.001*	*0.000*	*0.024*	*0.003*	*0.000*

The values are the means of three replicates with standard deviation (± SD). Different letters indicate within each column significant differences at p <0.05 (Tukey HSD test). IAA, Indole acetic acid; ABA, Abscisic acid; GA, Gibberellic acid; SA, Salicylic acid; JA, Jasmonic acid. T_0_: B0Cd0, T_1_: B0Cd1, T_2_: B0Cd2, T_3_: B0Cd3, T_4_: B1Cd0, T_5_: B1Cd1, T_6_: B1Cd2 and T_7_: B1Cd3. B0: No biochar, B1: 3% biochar, Cd0: No cadmium, Cd1: 50 mg kg^-1^, Cd2: 100 mg kg^-1^ and Cd3: 200 mg kg^-1^.

Statistical analysis results are italicized to distinguish them from average data.

## 4 Discussion

Cadmium (Cd) contamination threatens plant growth by disrupting physiological and biochemical processes, leading to reduced biomass and nutrient imbalances. Biochar, a carbon-rich byproduct of pyrolysis, has gained attention for its ability to improve soil properties and mitigate heavy metal toxicity, enhancing plant resilience. In our study examining the effects of biochar on the physiological, morphological, and biochemical properties of forage soybean under Cd stress, the findings revealed that Cd stress caused significant reductions in the growth parameters of forage soybean (**Table 1**). This outcome may be attributed to Cd accumulation disrupting the physiological and biochemical reactions of plants, ultimately leading to adverse effects on plant growth and morphology (Sgherri et al., 2002). Additionally, this situation could result from Cd accumulation in plants negatively impacting photosynthesis (Chen et al., 2011), nutrient distribution (Abu-Muriefah, 2008), and plant-water relations and aquaporin systems (Hatamian et al., 2019, 2020). Indeed, as observed in some studies, Cd has been reported to inhibit seed germination (Thamayanthi et al., 2011), hinder plant growth and development (Zhoa et al., 2021), and even cause plant mortality (Hsu and Kao, 2007). On the other hand, the results of our study indicated that biochar application significantly improved the growth and development of forage soybean under Cd stress (**Table 1**). These findings may be due to biochar’s ability to effectively reduce the phytotoxicity of heavy metals (O’Connor et al., 2018). Indeed, similar studies have reported that biochar, with its porous structure and large surface area, reduces Cd levels and positively impacts both growth and yield (O’Connor et al., 2018; Abbas et al., 2017), which supports the findings of our research. Zhang et al. (2021) reported that wheat grown in soil supplemented with new biochar improved plant height and dry weight while reducing cadmium content in the plant. The reason biochar has a good removal effect on cadmium in soil is due to its specific iron-containing functional groups and mineral crystal structure. Due to its high content of cellulose, hemicellulose, and lignin, hazelnut shell is considered a promising material for making biochar. These components help improve the biochar’s specific surface area, porosity, and carbonization degree (Bakisgan et al., 2009; Zhao et al., 2017). In the study, T_4_ (B1Cd0) as a biochar-only control improved the plant growth compared to the non-treated control T0 (B0Cd0) (**Table 1**). This could be attributed to improved soil physical, chemical, and biological properties with biochar (Zhu et al., 2022).

Adding biochar to soil has been shown in many studies to boost the productivity of soil microbes and significantly improve the makeup of microbial communities. Poplar bark and thiourea-modified poplar bark biochars improved the physicochemical properties and enzymatic activities of reclaimed soil at the mining site, thereby reducing Cd availability. Furthermore, these biochars reconstituted abundant and rare microbial communities in contaminated soils and were effective in improving the diversity, structure, and distribution patterns of bacterial and fungal communities (Zhu et al., 2022). Cadmium’s toxicity to organisms limits Cd removal by microorganisms. However, Chen et al. (2020a) determined that biochar’s pore structure protects bacterial cells from Cd stress, creating a positive feedback between P-rich biochar and Enterobacter sp., thus protecting bacteria by providing a P source to support microbial growth. Biochar can also make soil better by improving pH, air circulation, and water retention. These changes then lead to better growth patterns for soil microbes right around plant roots sugarcane bagasse-derived biochar (SBDB) effectively controlled Cd availability in contaminated soil (Haider et al., 2021). Its application markedly altered soil properties such as pH, soil organic matter (SOM), electrical conductivity (EC), and available phosphorus (AP). These modifications are likely responsible for the significant immobilization of Cd in the soil. Specifically, SBDB reduced the exchangeable form of Cd by converting it into less bioavailable fractions, resulting in considerable decreases in Cd concentrations within soybean tissues (Mohamed et al., 2019). Hazelnut shell-based biochar is an excellent resource for producing biochar. The waste product’s high cellulose content makes it a prime candidate for pyrolysis, yielding an economically valuable biochar (Gullap et al., 2024b). This biochar is particularly beneficial for soil health, as studies show it can improve aggregate stability, increase carbon content (Gullap et al., 2024b), and boost soil pH and organic matter (Mohamed et al., 2019). Environmental pollution highlights the need to re-evaluate and repurpose various waste materials, particularly those from agriculture. As an important renewable and cost-effective resource, agricultural waste can serve as a precursor for materials like activated carbon. For this study, we focus on hazel-nut shell-based biochar, a waste product with potential as a biochar. Unfortunately, this valuable carbon-rich and organic-matter-rich material is not currently being recycled in Turkey.

Cadmium inhibits enzymes such as δ-aminolevulinic acid, dehydrogenase, and proto chlorophyll reductase, which are crucial for chlorophyll synthesis and CO_2_ fixation. This inhibition leads to a reduction in chlorophyll synthesis and disrupts the metabolism of photosynthetic pigments (Cui et al., 2019), explaining why Cd decreased the chlorophyll content (chlorophyll-a, chlorophyll-b, and total chlorophyll) in forage soybean plants in our study. Furthermore, other studies have also reported that Cd stress increases reactive oxygen species (ROS) production in plants, leading to a decrease in leaf chlorophyll content and chlorophyll degradation (Yildirim et al., 2021; Ayhan et al., 2005; Loi et al., 2018). However, in our study, the addition of biochar to the soil significantly increased the chlorophyll content of forage soybean plants (**Table 2**). This improvement may be attributed to biochar’s ability to protect photosynthetic pigments and chloroplast structure, thereby enhancing the photosynthesis process (Choi et al., 2009; Kamran et al., 2020). In fact, several studies have shown that biochar application increases chlorophyll content in plants under Cd stress (Abbas et al., 2017; Yousaf et al., 2016). Cadmium, which has a high bio accumulative capacity and forms a stable metal complex with the chloride (Cl^-^) anion (Ghallab and Usman, 2007), is readily absorbed and accumulated by plants even at very low concentrations (Hadi et al., 2014). As seen in **Table 4**, the increase in Cd levels may have raised the Cd content in the plants. However, the biochar used in the study reduced the increased Cd content in forage soybean plants due to effects Cd, as also observed in several other studies (Akhtar et al., 2015; Gullap et al., 2024b).

Cadmium stress conditions caused the elevated H_2_O_2_ and MDA content in forage soybean plants (**Table 5**). Plant cell membranes are among the first casualties of stress. In this study, Cd stress in soybean seedlings led to increased MDA production, a marker of membrane lipid peroxidation. Stressors induce the formation of ROS, which play a central role in this membrane damage. ROS accumulation disrupts various cellular components, including membranes, chlorophyll, lipids, proteins, and DNA, ultimately leading to cell death (Yildirim et al., 2021). Plants treated with biochar exhibited lower levels of MDA and H_2_O_2_ under Cd stress conditions compared to untreated plants (**Table 3**). This aligns with previous findings showing biochar’s ability to mitigate ROS production induced by stress. Consistent with this, prior research has demonstrated that biochar application reduces the elevated MDA and H_2_O_2_ levels typically observed in stressed plants, likely by lessening the overall impact of stress conditions (Yildirim et al., 2021, 2022). In this study, biochar application decreased proline and sucrose levels in soybean seedlings subjected to Cd stress (**Table 5**). This reduction likely stems from biochar’s ability to alleviate the detrimental effects of Cd. This aligns with Dadasoglu et al. (2022), who also reported that biochar lowered sucrose and proline content in Cd stressed plants. Biochar application moderated the Cd-induced increase in antioxidant enzyme activity in soybean seedlings (**Table 6**). Consistent with our findings, studies have shown that biochar reduces the elevated antioxidant enzyme activity associated with stress. For instance, (Gullap et al., 2024a) demonstrated that biochar lowers ROS levels in salinity-stressed bean plants. Biochar has a high surface area and porosity, allowing it to adsorb and immobilize toxic compounds like heavy metals. This prevents the plant from absorbing them, thereby reducing the trigger for ROS production. Biochar can improve soil structure, water retention, and nutrient availability. A healthier soil environment reduces the plant’s overall stress, leading to less ROS generation. Because biochar reduces the initial stress and the resulting ROS overproduction, the plant’s need for a high-level enzymatic defense is diminished. The plant’s internal systems sense that the threat has been mitigated and, in response, downregulate the production and activity of antioxidant enzymes. Therefore, the biochar’s influence is indirect. It’s not a direct command to the plant’s genes to “turn down” enzyme production. Instead, it’s an environmental modification that creates a less stressful condition. The decrease in enzyme activity is a downstream effect, a sign that the plant is no longer in a state of high alert.

In plants, macro mineral elements play a role in regulating stomatal movements, cell wall formation, enzyme activities, and photosynthesis, while micro-nutrient elements are crucial for electron transfer, enzyme activity, cell proliferation, IAA synthesis, regulation of carbohydrate metabolism, and chloroplast structure and function (Kumar et al., 2021). In this study, it was found that the mineral content in soybean plants under Cd stress significantly decreased, likely due to the negative effects on photosynthesis, mineral uptake, and storage (Rizwan et al., 2016; Kinay, 2018; Zhang et al., 2019). Nowadays, biochar is widely used in many crops (such as corn, rice, spinach and beans) to reduce the impact of toxic heavy metals (Southavong et al., 2012). Indeed, as can be seen from **Tables 3** and **4** in this study, the addition of biochar under Cd stress increased the mineral content in forage soybean. This effect is attributed to the binding of Cd to the high cation exchange surfaces of biochar (Bian et al., 2014). A study conducted by Zhang et al. (2014) found that biochar, which is rich in N, P, K and other plant nutrients, was effective in reducing Cd rates in plants by competing with Cd, which is in line with the results obtained in our study. The study found that biochar increased nutrient uptake under both normal and stressed conditions. This suggests that, although no relevant measurements were made, the nutrients in the soil are largely available to the plant. Because studies have shown that biochar increases the nutrient holding capacity of the soil with both its own nutrient content and its physicochemical and biological properties, making it easier for plants to access nutrients (Hou et al., 2022). Biochar derived from different agricultural wastes (rice straw, wheat straw, acacia, and sugarcane bagasse) was found to reduce Cd uptake by sunflower shoots in the soil by up to 70% (Bashir et al., 2021). Similarly, another study reported that biochar application to bean and corn plants significantly increased the levels of N, P, K, Ca, Fe, Zn, Cu, and Mn in the plants (Inal et al., 2015; Dadasoglu et al., 2022). Biochar’s surface, rich in carboxylate and other ionizable functional groups, increases its cation exchange capacity upon oxidation (Liang et al., 2006). This enhanced capacity improves soil nutrient absorption, leading to greater plant nutrient use efficiency (Nguyen et al., 2017). Furthermore, biochar application enhances plant nutrient uptake and improves soil physical and chemical properties (Walter and Rao, 2015). Consequently, biochar positively influences plant physiology by improving soil physical, chemical, and biological properties, which in turn promotes root development (Zhang et al., 2013; Lu et al., 2025). A strong negative correlation between plant growth metrics (e.g., shoot/root dry weight, plant height) and the concentration of Cadmium (Cd) in the plant tissues would confirm that Cd stress is indeed inhibiting growth. A decrease in Cd content in biochar-amended plants would directly correspond to an increase in their growth, proving that biochar’s primary benefit is reducing Cd uptake.

The study shows detailed analysis of how hazelnut shell biochar affects key phyto-hormones under Cd stress. It found that Cd stress decreased the content of growth-promoting hormones (IAA, GA, SA, CK, zeatin, and JA) and increased the stress-related hormone ABA. The application of biochar reversed this trend, increasing the levels of growth-promoting hormones and decreasing ABA content, which helps explain the improved growth and resilience of the soybean plants. Plant growth hormones, critical for growth and development at low concentrations are essential for stress adaptation and act as key defense mechanisms against abiotic stressors, including metal toxicity (Machackova et al., 2008). Key hormones such as indole-3-acetic acid, gibberellins, cytokinins, salicylic acid, ethylene, abscisic acid, jasmonic acid, brassinosteroids, and strigolactones are known to modulate plant responses to Cd stress (Bari and Jones, 2009; Asgher et al., 2015). Indeed, as observed in this study, under Cd stress conditions, the ABA content in forage soybean seedlings increased, while the levels of IAA, GA, SA, CK, zeatin, and JA decreased (**Table 7**). The increase in ABA, which is known to play an active role in plant stress responses and to rise under stress conditions (López-Climent et al., 2011), supports the findings of our study, as ABA is considered a key internal signal against abiotic stress factors (Javid et al., 2011). Furthermore, studies (Yue et al., 2016; Vishwakarma et al., 2017) have reported that GA_3_, ABA, JA, and SA mediate plant responses to abiotic stress, with ABA, SA, and IAA playing significant roles in plant responses to metal stress (Hong-Bo et al., 2010). Additionally, studies on plants such as rice (Rahman et al., 2016), tomato (Alves et al., 2017), corn (Perez Chaca et al., 2014), and fenugreek (Ahmad Dar et al., 2015) have shown that the response to Cd stress is mediated through plant growth hormones. However, in this study, it was found that biochar, which positively affects plant development and can adsorb organic molecules (Eo et al., 2018), reduced the negative effects of Cd stress on growth hormones (**Table 7**). This positive effect may be attributed to biochar’s high adsorption capacity, resulting from its negative surface charge, high charge density, and large surface area of approximately 500 m^2^g^-1^ (Lorenz and Lal, 2014). This increase in soybean plant growth hormones caused by biochar also indirectly contributed to increased plant root and shoot development. Yang et al. (2025) determined that biochar positively affects root growth by affecting root hormone levels and plays a role in regulating nitrogen metabolism. In beans, levels of stress hormones such as ABA, ACC, and JA, which increase during salt stress, were reduced by biochar application, while growth-promoting hormones such as IAA were increased. This suggests that the plant is under less stress with biochar and does not require an excessive defense mechanism (Farhangi-Abriz and Torabian, 2018). The decrease in stress hormones and the increase in growth hormones due to biochar addition to the soil may be associated with a decrease in Cd uptake into plant tissues, as noted by Farhangi-Abriz and Ghassemi-Golezani (2022) in their own study. A key finding is the clear reduction in plant Cd uptake in the biochar-amended treatments. This reduction in heavy metal stress likely had a profound indirect effect on the plants’ hormone regulation. Cadmium is known to disrupt the synthesis and signaling of key growth-regulating hormones, often leading to a hormonal imbalance that inhibits growth (Lorenz and Lal, 2014). Therefore, the observed increase in growth-promoting hormones (e.g., auxins) and decrease in stress-related hormones (e.g., abscisic acid) can be interpreted as a physiological response to the alleviation of cadmium toxicity. Beyond direct stress mitigation, the addition of biochar can have broader ecological and physiological trade-offs. Biochar can alter soil water-holding capacity, nutrient cycling, and microbial communities, all of which are known to indirectly influence plant hormone levels (Gullap et al., 2024b; Zhao et al., 2025). For instance, an improved soil water status could lead to increased cytokinin synthesis, a hormone critical for cell division and shoot growth. Future research should employ a more detailed time-course analysis to track the dynamic changes in phytohormones and directly link them to both the reduction in heavy metal stress and other biochar-induced changes in the soil environment.

The Cd immobilization mechanism involves processes such as adsorption, precipitation, reduction, ion exchange, surface complex formation, hydrogen bonding, π–π interactions, and pore filling. In recent years, it has been noted that biochar can be further enhanced by processing and modifying it. Indeed, it has been observed that modified biochar, thanks to its large specific surface area, porous structure, and various functional groups and active sites on its surface, can enhance Cd immobilization in soil (Rahim et al., 2022). While our results show a significant reduction in mobile Cd, the precise immobilization mechanisms (e.g., precipitation, specific adsorption) remain speculative due to the absence of direct spectroscopic or sequential extraction data in this study. The immobilization of Cd by biochar is a crucial mechanism for mitigating its environmental risks. The application of biochar significantly reduces the mobility and bioavailability of Cd in soil (Rahim et al., 2022; Qi et al., 2023).

However, it is thought that the biochar used in this study, especially thanks to its humic substances, shows similar effects and supports Cd immobilization and therefore plant growth. Hazelnut shells are ideal for creating microporous biochar due to their high cellulose content, a valuable resource for pyrolysis (Zhao et al., 2017). This biochar exhibits enhanced adsorption capacity due to its increased surface area and porosity. Furthermore, hazelnut shells con-tribute to ecologically safer biochar by reducing heavy metal content (Chen et al., 2020b).

Although gene expression analyses have not been conducted, the activation of various genes may also play a role in the Cd stress mitigation effect of biochar. One study indicated that biochar alleviated Cd stress in melon by inhibiting Cd transfer, increasing resistance to Cd stress through activation of the phenylpropanoid pathway, and overexpression of stress-related genes (11 genes of the enylpropane pathway, cytochrome P450 family protein genes, and WRKY transcription factor genes) (Cheng et al., 2023). The effects of biochars produced from different agricultural wastes on various plants under Cd stress are similar to the hazelnut biochar used in our study. Rice straw and wheat straw biochars have been shown to be effective in sunflower growth under Cd stress (Bashir et al., 2021), tobacco stem biochar application can repair soils by converting Cd to low-hazardous forms and increase tobacco productivity (Yu et al., 2021), and biochar derived from fruits and vegetables (orange, potato) is an effective tool for reducing stress in maize (Anwar et al., 2024). Reutilizing agricultural waste, like hazelnut shells, is crucial for environmental sustainability. These wastes serve as cost-effective activated carbon precursors. However, in Turkey, hazelnut harvest waste, a rich carbon source and organic amendment, is unfortunately not adequately recycled. This study is the investigation into the use of biochar derived specifically from hazelnut shells to mitigate cadmium (Cd) toxicity in forage soybean. While previous research has explored biochar from various sources for heavy metal remediation, studies on biochar produced from hazelnut shells are noted as being limited. This is particularly relevant as Turkey is the world’s primary producer of hazelnuts, and the shells are a significant agricultural waste product that is not currently being recycled.

## 5 Conclusions

In conclusion, our findings demonstrate that biochar amendment shows promising potential for mitigating cadmium toxicity in a controlled laboratory setting. The observed reduction in plant Cd uptake and the improved plant growth provide a valuable preliminary foundation. However, we must stress that these findings should be interpreted with caution. The absence of field validation and the limited experimental scope make it premature to claim direct applicability for agricultural or environmental remediation purposes. Future research must focus on validating these results in a field setting under varying environmental conditions and with a wider range of biochar properties. Furthermore, a comprehensive analysis of the potential ecological trade-offs, such as nutrient immobilization or alterations to the soil microbial community, is essential for a complete risk-benefit assessment of biochar use.

## Data Availability

The raw data supporting the conclusions of this article will be made available by the authors, without undue reservation.
